# Identification of novel transcriptional regulators involved in macrophage differentiation and activation in U937 cells

**DOI:** 10.1186/1471-2172-10-18

**Published:** 2009-04-02

**Authors:** Young-Sook Baek, Stefan Haas, Holger Hackstein, Gregor Bein, Maria Hernandez-Santana, Hans Lehrach, Sascha Sauer, Harald Seitz

**Affiliations:** 1 Department of Vertebrate Genomics, Max-Planck-Institute for Molecular Genetics, 14195 Berlin, Germany; 2 Department of Computational Molecular Biology, Max-Planck-Institute for Molecular Genetics, 14195 Berlin, Germany; 3 Institute of Clinical Immunology, University Hospital, 35392 Giessen, Germany; 4 Department of Rheumatology and Clinical Immunology, Charité University Medicine, 10117 Berlin, Germany

## Abstract

**Background:**

Monocytes and macrophages play essential role in innate immunity. Understanding the underlying mechanism of macrophage differentiation and the identification of regulatory mechanisms will help to find new strategies to prevent their harmful effects in chronic inflammatory diseases and sepsis.

**Results:**

Maturation of blood monocytes into tissue macrophages and subsequent inflammatory response was mimicked in U937 cells of human histocytic lymphoma origin. Whole genome array analysis was employed to evaluate gene expression profile to identify underlying transcriptional networks implicated during the processes of differentiation and inflammation. In addition to already known transcription factors (i.e. MAFB, EGR, IRF, BCL6, NFkB, AP1, Nur77), gene expression analysis further revealed novel genes (i.e. MEF2, BRI, HLX, HDAC5, H2AV, TCF7L2, NFIL3) previously uncharacterized to be involved in the differentiation process. A total of 58 selected genes representing cytokines, chemokines, surface antigens, signaling molecules and transcription factors were validated by real time PCR and compared to primary monocyte-derived macrophages. Beside the verification of several new genes, the comparison reveals individual heterogeneity of blood donors.

**Conclusion:**

Up regulation of MEF2 family, HDACs, and H2AV during cell differentiation and inflammation sheds new lights onto regulation events on transcriptional and epigenetic level controlling these processes. Data generated will serve as a source for further investigation of macrophages differentiation pathways and related biological responses.

## Background

The mammalian innate immune system, comprised of macrophages, provides a front line of defense against pathogens. Infection or dysfunction of macrophages results in clinical situations like septic shock or chronic inflammatory disease such as atherosclerosis and rheumatoid arthritis. Under normal steady-state conditions, monocytes migrate randomly from blood to various organs or body cavities and differentiate into macrophages through coordinate expression of numerous genes. During a local infection, migration of blood monocytes into the inflamed tissues is accelerated, and subsequent differentiation into macrophage occurs rapidly [1]. Upon the entry of microorganisms, conserved structures found in a broad range of pathogens are recognized by toll-like receptors (TLRs) of macrophages. Phagocytic macrophages are activated to produce inflammatory cytokines (i.e. TNFalpha) and chemokines (i.e. IP10). Signaling pathways triggered by individual TLRs involve recruitment of adaptor molecules (i.e. MyD88) and autocrine production of IFNbeta. Two distinct cascades, MyD88 dependent and MyD88 independent (IFNbeta mediated), lead to activation of immuno-regulatory transcription factors (i.e. IRFs, STATs, NFkB, AP1) to modulate inflammatory gene expression [2].

Transcriptional regulators play important roles during developmental processes, and were described in a variety of well-studied differentiation systems. During the process of hematopoietic differentiation of monocytes, functions of transcription factors encompass up regulation of genes specific to the lineage and repression of lineage-inappropriate genes by forming distinct protein complexes [56]. Similarly, cell-type-specific gene expression during macrophage differentiation and function is a result of complex transcriptional regulation by the interplay between endogenous transcription factors and external signals, and also by the networks of individual transcription factors. Whatever other mechanisms can modulate their activity, expression of specific transcription factors during differentiation process is likely to be regulated transcriptionally. Several transcription factors including MAFB, EGRs, NFkB, AP1, IRFs and BCL6 that control various stages of macrophage development or function have already been identified [4,5]. However, only a genome-wide expression study will be able to identify all transcriptional regulators, gene regulatory networks and help to understand molecular mechanisms underlying relevant immune disease.

Furthermore, the role of chromatin structure in gene regulation of immune system has been increasingly studied. In activated macrophages stimulated by LPS, the transcriptional response has been attributed to the expression and function of chromatin remodeling genes, mainly related to histone modifications [6-8]. Timely coordinated relationship regarding transition of chromatin structure, nucleosome remodeling and transcription factors are known to be important [9]. Under such a circumstance, it is of utmost importance to study genome-wide gene expression changes in model systems that make it feasible to continue investigating and dissecting underlying molecular mechanisms.

The use of a model system circumvents many problems including available cell numbers, high cost and tedious procedure, generally encountered while having to work with primary blood cells. However, the validity of any single myeloid model system has never been fully verified. Among several myeloid cell lines (HL60, THP-1, Mononomac-1, U937), U937 cells and THP1 cells are the most widely used models for investigating monocytic differentiation and subsequent biological functions of differentiated cells [10,11]. In contrast to THP-1 cells at less mature stage due to their blood leukemic origin, U937 cells of histocytic lymphoma origin are arrested in a more advanced stage of differentiation (promonocyte/monocyte). Various stimuli (i.e. DMSO, Retinoic acid, VitD3, PMA, IFNgamma), either alone or in combination, have been attributed to induce their terminal differentiation into monocytes or mature macrophages. Upon differentiation, U937 cells acquire a large repertoire of macrophage function through the concerted expression of numerous genes. Differentiated U937 cells can be further stimulated with LPS (*E. coli *Lipopolysaccharide) to mimic inflammatory response of activated macrophages.

In this study, we employed the U937 model system combined with a cRNA hybridization-based whole genome array to analyze genome-wide expression patterns during monocyte-macrophage differentiation and LPS-responsive activation of differentiated cells. The identification of novel transcriptional regulators intensifies directions for further investigation of the molecular mechanism involving common regulatory networks and epigenetic mechanisms.

## Results

### U937 cells were differentiated into monocyte and macrophage-like cells

U937 cells have been widely used as a model to investigate a variety of biological processes related to monocyte and macrophage function. Here, we used either the hormonally active form of Vitamin D3 (1, 25-dihydroxyvitamin D3) to differentiate U937 cells toward monocytes, or PMA (phorbol 12-myristate 13-acetate) to induce differentiation into a macrophage-like phenotype. Both PMA and VitD3 were known to induce cell cycle arrest prior to proceeding into differentiation stage. PMA-treated cells exhibit apparent growth arrest already 6 hrs after the addition of PMA and almost 100% adherence of cells after 24 hrs. Cells then tend to loose adhesiveness gradually afterwards. Growth arrest phenotype was much less prominent with VitD3-treated cells that continued moderate proliferation even after addition of the reagent (data not shown). Cells differentiated for 24 hrs by each inducer were used for further analysis and LPS stimulation.

Fluorescence activated cell-sorting (FACS) was carried out to monitor two surface proteins characteristic of differentiated U937 cells (data not shown): (1) CD14, serving as a marker for both monocyte and macrophage, is involved in LPS recognition and facilitating LPS contact with TLR4 receptor; (2) CD11b, serving as a macrophage marker, is involved in phagocytosis of bacteria. CD14 expression was increased after VitD3 differentiation, yielding the characteristics of monocyte-like cell type. This result is also in accordance with mRNA expression, and is certainly indicative of enhanced sensitivity of these cells to LPS stimulation [12]. CD11b expression was increased only after PMA inducement, indicating macrophage-like differentiation, meanwhile no CD14 induction was observed with these cells.

**Figure 1:**
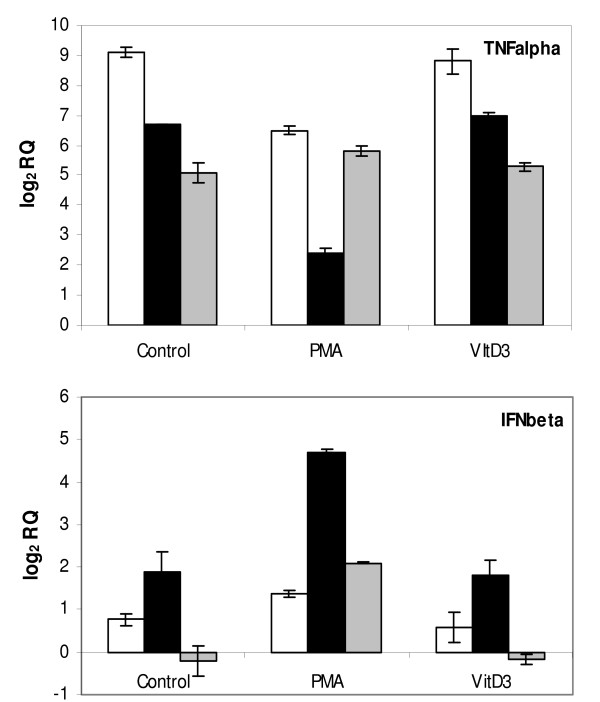
**Differential mRNA accumulation of TNFalpha and IFNbeta**. Only PMA differentiated cells, representatives of macrophages, exhibit distinct biphasic behavior of TNFalpha accumulation (2 and 22 hrs) and time dependent up regulation (10 hrs and 22 hrs) of IFNbeta. Error bars refer to technical variation. Relative expressions were represented as log RQ (base 2) in which RQ (relative quantification) = 2^-ddCt^. Different duration of LPS stimulation was indicated by white bar (2 hrs), black bar (10 hrs for IFNbeta, 12 hrs for TNFalpha) and gray bar (22 hrs) on the graph.

According to well-known molecular mechanisms underlying macrophage activation, TNFalpha is a gene product of MyD88-dependent pathway (common to both monocytes and macrophages) while IFNbeta is an autocrine product destined to launch MyD88-independent pathway specific for macrophages [2]. In the case of sepsis, persisting and systemic release of TNFalpha by activated macrophages can cause organ failure and death as a result of septic shock. Inevitably, it would require tight regulation. In order to demonstrate successful differentiation leading to activated macrophages, mRNA accumulation of TNFalpha and IFNbeta was analyzed by real-time PCR. The result from stimulation of PMA and VitD3 differentiated cells with 10 μg/ml LPS is shown in Figure 1. Immediate expression (2 hrs) of TNFalpha was common to all samples (control, VitD3, PMA). With control U937 cells and VitD3-differentiated cells, decreasing production of TNFalpha mRNA was observed across the later period of stimulation (12 ~22 hrs). PMA differentiated cells shows a dramatically decreased TNFalpha expression after 12 hrs but increased again at 22 hrs. This distinct biphasic behavior is a manifestation that macrophage-like cells possess an intrinsic 'automatic-bridle' system to modulate production of this cytokine in a time dependent manner. Stimulation at the lower concentration of LPS (2 μg/ml) showed the same pattern except that the biphasic behavior was extended up to 48 hours. Time dependent mRNA accumulation of IFNbeta was also characteristic for PMA differentiated cells representative of macrophages. Initially, a moderate increase of mRNA was observed (at 2 hrs). Afterwards, further increase was prominently induced (10 hrs). By the time of 22 hrs, the mRNA level was maintained at the level comparable to initial stage. On the contrary, control U937 cells and VitD3 differentiated cells manifest much less prominent accumulation of IFNbeta after both 2 and 10 hrs, and are completely devoid of it by the time of 22 hrs. It indicates that these cells are inefficient (during earlier period) and finally lacking (during later period) in IFNbeta induced MyD88-independent pathway.

### Differential gene expression of U937 cells manifests functional macrophage phenotype

Whole transcriptome expression profiling was performed with two biological replicates and two technical replicates from each of seven different samples (control, PMA 6 hrs, PMA 12 hrs, PMA 24 hrs, PMA 32 hrs, LPS, VitD3) while satisfying each set of replicates on separate slides. To evaluate the consistency of data across biological or technical replicates, correlation coefficients were calculated. In all cases, the correlation between biological replicates and also technical replicates were higher than 0.98 (see Additional file 1). During PMA differentiation, there was a shift of correlation coefficients from 0.961 (after 6 hrs) to 0.919 (after 32 hrs), indicating 3.9% to 8.1% of gene features contained on the array were differentially regulated. There appeared only 0.6% (0.925 ~0.919) of change between 24 hrs and 32 hrs of PMA differentiation. This shows that the cells were already reached a state of full differentiation after 24 hrs. It is noteworthy that additional 3.8% of features were promptly induced within 2 hrs of subsequent LPS stimulation.

**Figure 2:**
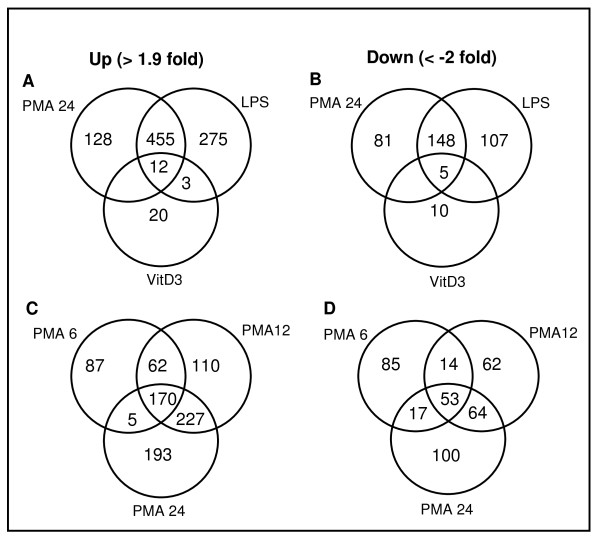
**Gene expression profile**. Shown are numbers of up regulated (A) and down regulated (B) genes after PMA, VitD3 differentiation and LPS stimulation (2 hrs). Numbers of differentially regulated genes at different time points (6, 12, 24 hrs) during PMA differentiation are shown in C (for up regulation) and D (for down regulation).

Gene expression of differentiated or stimulated cells was evaluated in comparison to undifferentiated U937 cells. Differential gene expression was considered 'significant' when the normalized intensity ratio of sample vs control was >1.9 (up) or <-2 (down), respectively (Figure 2A–D). At 6 hrs of PMA differentiation, 324 genes were up regulated and 169 genes down regulated. After 12 hrs, 337 genes were additionally up regulated while 126 genes were down regulated. By the time of 24 hrs, total 595 genes were up regulated and 324 genes down regulated. After 2 hrs of subsequent LPS stimulation, 278 additional genes were up regulated while 107 additional genes were down regulated. With VitD3 differentiation, only 35 genes were significantly up regulated while 15 genes down regulated. The complete list of gene expression profiling is available in Additional file 2.

Top 50 most highly up regulated genes, sorted by 24 hrs of PMA differentiation, are shown in Table 1. Among them are several genes well known to be related with differentiation process as well as monocyte and macrophage functions. This involves proteins known to be involved in the differentiation process (CDK inhibitor p21), cytokine transporter like A2M, chemokines like CCL3 and IP10 as well as matrix metalloproteinase protein such as MMP7 and MMP9 involved in the proteolysis of structural and adhesive matrixes.

**Table 1:** List of top 50 up regulated genes (sorted by PMA 24 hrs)

		Illumina array fold change					
		
Ref.Seq.	Gene Symbol	PMA6	PMA12	PMA24	PMA32	LPS	VitD3
NM_014220	HL6	20.6	88.5	96.3	96.5	128.2	1.1
NM_005368	MB	4.3	27.8	90.5	110.5	96.2	7.7
NM_000582	BNSP	7.6	31.1	84.1	118.1	110.9	1
NM_021796	PLAC1	1.8	11.8	34.6	39.1	34.3	-1.1
NM_002423	MMP7	1.9	2.7	29.9	61.4	36.5	-1.4
NM_002922	RGS1	15.9	25.6	28.6	26	29.9	-1.1
NM_018993	RIN2	9.5	18.2	23.2	18.9	14.4	1
NM_001955	EDN1	42.9	22.9	21.2	22.9	85.4	1
NM_000265	NCF1	3.4	11.2	18.6	21	17.8	3.8
NM_001332	CTNND2	1.5	4.9	15.7	20.4	12.8	1.1
NM_016270	KLF2	15.5	11.6	13.9	15.2	9.8	-1.3
NM_002167	ID3	1.8	3.4	11.9	6.2	38.9	2.7
NM_002203	CD49B	2	7	10.5	9.3	6.9	1
NM_000014	A2M	4.1	7.4	10.4	11.1	11.5	1.1
NM_001401	EDG2	4.4	9.2	10.3	8.8	6.6	-1.8
NM_152591	CCDC63	2.5	7.1	9.9	9.8	10.6	-1.1
NM_030820	COL20A1	1.2	2.8	8.8	7.7	7.4	-1.1
NM_004994	MMP9	2.4	6	8.4	10.1	8.3	-1.2
NM_004926	BERG36	6.5	8.1	8.2	7.7	9.9	-1.4
NM_001200	BMP2	15.2	13.6	8.1	4.1	10.3	-1.2
NM_015271	RNF86	2.6	5.5	8.1	10	7	-1
NM_021952	ELAVL4	7.3	8.1	8	7.9	6.8	-1.1
NM_001706	BCL6	7.1	8.3	7.6	7.6	19.5	1.6
NM_001565	CXCL10	1.4	1.5	7.5	12.6	23.4	-1
NM_005627	SGK	3.4	4.9	7.5	6.6	13	-1
NM_007351	ECM	3.3	7.2	7.3	8.4	8	1.1
NM_206827	RASL11A	1.2	4.3	7.2	6	4.5	1.2
NM_002983	CCL3	8.9	8.9	7.1	8.2	197.3	1.4
NM_003294	ALPHAII	2.4	4.6	7.1	9.4	8.6	1.1
NM_153370	PI16	1	1.6	6.9	10.3	6.9	1
NM_003986	BBH	1.7	3	6.8	9.8	6.6	-1.2
NM_025113	C13ORF18	2.5	4.7	6.7	5.3	14.8	1.3
NM_005449	TOSO	3.7	8.4	6.4	6	7	1.4
NM_000389	P21	2.4	4.2	6.1	6.1	11.8	1.3
NM_001001437	CCL3L3	8.5	7.8	6	6.1	187	1.2
NM_001268	CHC1L	1.6	3.4	6	6.8	4.7	1.1
NM_206939	CD20L4	-1.2	2.8	5.9	8.3	6.9	-2
NM_004615	A15	1.2	3.5	5.8	6.3	4.8	-1.3
NM_016134	PGCP	1.4	3	5.7	7.1	5.4	-1.1
NM_005211	CD115	2.2	5.6	5.6	4.8	5.1	1.7
NM_147130	LY117	2.8	5.3	5.6	4.5	5.3	1.2
NM_013445	GAD	1.1	3.9	5.6	5.9	5	-1.2
NM_007079	PRL3	1.6	3.9	5.6	7.9	7.1	-1.2
NM_032199	ARID5B	7	5	5.5	5.7	6.6	-2.1
NM_001257	CDH13	1	2.7	5.5	5.6	5.1	-1.2
NM_001017998	GNG10	2	4.3	5.5	7.4	5.3	1.5
NM_005842	HSPRY2	4.3	5.8	5.4	5.2	4.1	-1
NM_016257	HLP4	1.2	2.7	5.4	7	5.3	1.2
NM_052815	DIF2	6.8	5.1	5.3	4.7	157.7	1.1
NM_014862	ARNT2	2.5	5.4	5.3	4.8	5.3	-1

The column designation indicates different time points of differentiation with PMA (6 hrs, 12 hrs, 24 hrs and 32 hrs), VitD3 (24 hrs) and subsequent activation of PMA differentiated cells (24 hrs) by LPS (2 hrs). The numbers denote fold change after normalization of Illumina array data. The order of genes was sorted by PMA 24 hrs sample in descendent manner.

### Temporal pattern of array gene expression can be categorized

**Figure 3:**
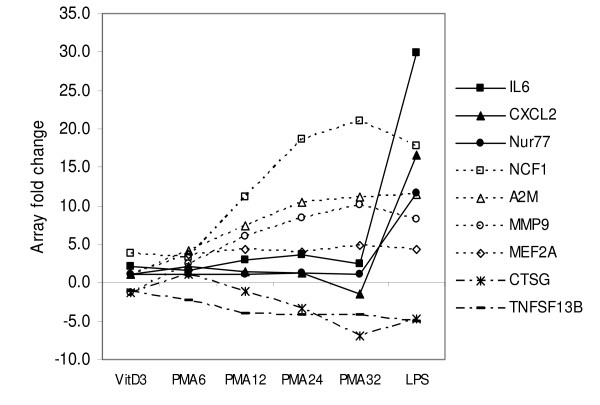
**Temporal patterns of gene expression**. Shown are examples of three distinct groups: outstanding up regulation upon LPS stimulation (IL6, CXCL2, Nur77), constant up regulation (NCF1, A2M, MMP9, MEF2), and constant down regulation (CTSG and TNFSF13B) during macrophage differentiation period.

Large scale comparisons of differential gene expression profiles between different experimental conditions (for example, different time points of differentiation and activation) have potential to identify co-regulated and/or inter-related genes. Such categories reflect their functional relationships and can help to identify common transcriptional control mechanisms. Transcription factors in such groups of genes may be responsible for regulation of the genes in the same group. Beside genes not affected, three distinct groups could be categorized. Nine examples of time dependent gene expression were illustrated in Figure 3. IL6 (cytokine), CXCL2 (chemokine) and Nur77 (transcription factor) represent a group of genes that are highly up regulated only after LPS-responsive activation. NCF1, A2M, MMP9, all of which related to macrophage function, and transcription factor MEF2A represent a second group of genes whose expression was constantly up regulated during differentiation period and also activation. CTSG and TNFSF13B, represent a third group of genes specifically down regulated during macrophage differentiation. Both proteins are related to functions in neutrophils and B cells respectively.

### Validity of expression analysis was verified by real timePCR

Among 58 selected genes validated by real time PCR (Table 2), were cytokines and chemokines (TNFalpha, IL1A, IL1B, IL6, IL8, CCL3, CCL4, CXCL2, IP10), inflammatory effector molecules (MMP7, MMP9, PLAU, C3, A2M, NCF1, LAMB3, LL37, CD38), surface antigens (CD11b, CD14, CD69, CD83), signaling molecules (MyD88, TLR2, SGK, JAK3) and transcription factors.

**Table 2:** Validation of array data by real-time PCR

		Illumina array fold change			Real time PCR			
					
					Ct		RQ fold *	
		
Ref.Seq	Gene Symbol	PMA24	LPS	VitD3	Cont	PMA24	LPS	VitD3
Cytokines and Chemokines								
NM_002983	CCL3	7.1	197.3	1.4	26	27.9	776	1.6
NM_000594	TNFa	3	121.4	1.6	29	13.9	119.4	1.1
NM_000584	IL8	3.7	82.9	2.3	25	1.2	222.9	3.2
NM_000575	IL1A	1.1	98.7	-1	35	5.7	2195	2.3
NM_000576	IL1B	2.9	75.2	1.1	26	2.5	415.9	0.7
NM_004591	CCL20	-1	47.6	1.1	30	2.1	724.1	0.9
NM_000600	IL6	3.7	29.9	2.1	28	4.6	157.6	3.5
NM_001565	IP10	7.5	23.4	-1	30	97	222.9	0.7
NM_002089	CXCL2	1.2	16.5	1.1	28	0.4	48.5	1.7
NM_002984	CCL4	1.1	1.7	1.1	35	52	80,000	1.9
Surface molecules								
NM_004233	CD83	1.8	10.4	-1.1	23	3.2	9.8	0.6
NM_001781	CD69	1.4	4.1	-1.4	21	3.2	4.9	0.9
NM_000632	CD11b	3.3	3.1	2.7	24	7.5	3	2.6
NM_000591	CD14	-1	1.2	22.4	29	1.1	1.1	45.3
Signaling and effector molecules								
NM_002423	MMP7	29.9	36.5	-1.4	28	512	181	0.6
NM_002658	PLAU	1.3	19.9	-1	28	1.1	5.3	0.6
NM_000265	NCF1	18.6	17.8	3.8	29	32	39.4	2.6
NM_005627	SGK	7.5	13	-1	22	10.6	52	1.6
NM_000389	p21	6.1	11.8	1.3	25	42.2	42.2	1.1
NM_000014	A2M	10.4	11.5	1.1	30	52	32	3.2
NM_004994	MMP9	8.4	8.3	-1.2	30	21.1	9.2	1.1
NM_000228	LAMB3	2.9	6.5	8.5	28	32	19.7	64
NM_002468	MYD88	4.5	4.2	1.4	27	27.9	7.5	1.5
NM_000064	C3	1.8	2.3	-1	27	119.4	42.2	1.2
NM_003264	TLR2	1.4	1.6	1.1	31	11.3	3.2	2
NM_000215	JAK3	1.5	1.7	1.1	18	5.7	4.9	1.4
NM_004345	LL37	-1.2	-1.3	32.1	30	1.3	0.8	84.4
NM_001775	CD38	-1.9	-2	5.9	22	1.1	0.2	7.5
NM_001911	CTSG	-3.4	-4.8	-1.3	21	0.03	0.01	0.6
NM_006573	TNFSF13B	-4.3	-5.1	-1.2	22	0.3	0.1	1.9
NM_000250	MPO	-8.3	-9.6	-1.1	17	0.1	0.03	1.1
Transcription factors/Histone modifiers								
NM_005384	NFIL3	3.1	4.9	-1.3	27	9.8	78.8	3.2
NM_000321	RB1	2.2	1.8	1	19	3	1.1	-0.2
NM_005461	MAFB	3.3	35.8	1.2	27	17.1	18.4	-1.1
NM_005587	MEF2A	4	4.4	1.1	19	9.2	4.3	1.1
NM_002397	MEF2C	1.8	1.9	-1.2	19	8	4.3	1.6
NM_005920	MEF2D	2.1	7.9	-1.1	26	1.1	3	0.5
NM_021958	HLX1	2.2	2.3	-1	23	10.6	3.5	1.2
NM_021999	BRI	2.6	2.2	-1.2	22	8.6	3.7	1.2
NM_005474	HDAC5	NA			24	4.9	4	0.8
NM_012412	H2AV	NA			19	2.6	1	1.3
NM_173158	Nur77	1.2	11.7	1.1	35	1.1	2.3	1
NM_001706	BCL6	7.6	19.5	1.6	25	78.8	34.3	8
NM_001964	EGR1	4.2	13.6	1.1	27	14.9	1.2	1.4
NM_005252	FOS	2.4	3.3	-1.5	26	32	2.6	0.9
NM_002167	ID3	11.9	38.9	2.7	30	13	18.4	8
NM_006084	IRF9	2.5	2.8	1	25	214.5	5.3	1.4
NM_000399	EGR2	5.3	27.3	1.1	28	5.3	4.3	2.5
NM_016270	KLF2	13.9	9.8	-1.3	26	32	97	0.5
NM_021784	FOXA2	4.9	2.9	-1	30	73.5	16	0.3
NM_005524	HES1	2.8	2.8	1.2	32	27.9	64	0.8
NM_004235	KLF4	1.5	1.7	-1	27	3.7	3	1.9
NM_002698	OCT2	2.1	3.3	-1.2	26	14.9	7	0.4
NM_030756	TCF7L2	3	2.8	-1.2	21	8.6	5.3	0.5
NM_004030	IRF7	2.2	2.6	-1.3	26	13	4.6	1.2
NM_014707	HDAC7	3.2	2.5	-1.2	22	29.9	16	0.7
NM_003884	PCAF	2	1.8	-1.2	25	3.7	1.4	0.8
NM_005919	MEF2B	1.3	1.4	1.3	27	6.5	0.4	0.8

58 genes representative of cytokines, chemokines, signaling molecules, transcription factors and histone modifiers were selected. NA (none applicable) denotes data not available from array experiment. *RQ = 2^-ddCt ^in which ddCt = dCt_(samples) _- dCt_(control U937 cells)_. dCt denotes Ct value normalized to GAPDH.

The fold change of greater than 20 from the array correspond to a factor of greater than 100 RQ fold (relative quantification, in which RQ = 2^-ddCt^) based on real time PCR (>ddCt -7). Likewise, array fold change between 10 and 20 usually fell within the range of 10 to 50 RQ fold while low fold change around 2 or 3 were equal to various range from less than 5 up to 30 RQ fold. Taking an example of LPS stimulated sample, confirmation rate of up or down regulation by real time PCR result was 100%. With PMA samples, a few cases of inconsistency, as with IL8, IL1A and CCL4, were observed

### Identification of transcriptional regulators

Transcriptional regulators already known to be involved in monocytic-lineage differentiation (i.e. EGR1, EGR2 and MAFB) are constantly up regulated during the differentiation process and subsequent activation (see Additional file 3). Previously unknown factors (i.e. BRI, HLX1, TCF7L2, MEF2 family) were also shown to be significantly and constantly up regulated. Of the most interesting new identified transcription factors are proteins belonging to the MEF2 (myocyte enhancer factor 2) family. Moreover, a target gene (i.e. Nur77) of MEF2 during T cell apoptosis and postsynaptic differentiation were also up regulated. This is in line with previous reports [13-15]. Among the population of transacting proteins identified, several factors acting as transcriptional repressors (ID3, BCL6, NFIL3) and histone modifying proteins (HDAC5, HDAC7/9) were verified. Histone deacetylases (HDACs) were previously implicated as key molecules to repress production of pro-inflammatory gene expression, thus prevent excessive inflammatory responses [16]. KLF2 and KLF4 have been recently described as critical regulators controlling monocyte differentiation and proinflammatory activation [17,18]. Both factors are validated to be up regulated by real time PCR (Table 2). This together with up regulation of KLF6 suggests importance of kruppel-like family of transcription factors (KLFs) in the differentiation and function of macrophages. Upon LPS stimulation, NUR77, FOS, JUN, STAT3, IRF family, NFkB family and SMAD family show significant up regulation, thus confirm their classical role in macrophage function of inflammatory response. One of the main physiological roles of NFkB family is in the development and functioning of the immune system by regulating transcription of cytokines and antimicrobial effectors as well as genes that regulate cellular differentiation, survival and proliferation [19]. Consistently, RELB, cREL, NFkB1 and NFkB2 are significantly induced upon LPS stimulation, thus confirming the validity of U937 model system.

### Cross validation between U937 cells and primary blood monocytes

**Figure 4:**
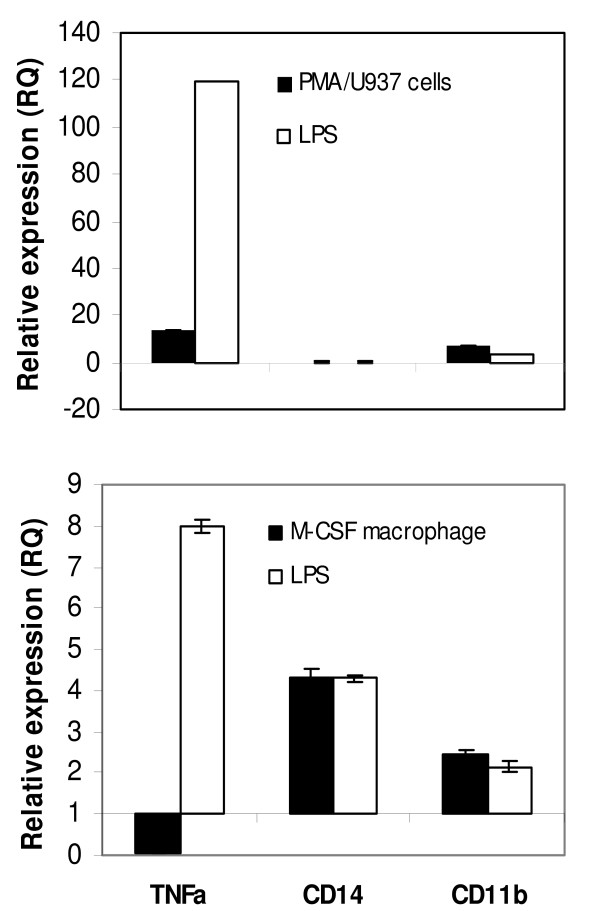
**Comparison of real time PCR result upon differentiation and activation between primary monocytes and U937 cells**. (Upper panel) Up regulation of CD11b alone but not of CD14 was observed with U937 cells differentiated with PMA. Prominent induction of TNFalpha after LPS stimulation was seen with both cell types. (Lower panel) Up regulation of CD14 and CD11b surely indicates maturation and activation of primary macrophages (M-CSF macrophages). Down regulation of TNFalpha upon macrophage differentiation reflects intrinsic abundance of mRNA with *in vivo *monocytes on the contrary to U937 cells. Mature macrophage stage was indicated by black bar (denoted as PMA/U937 cells or M-CSF macrophages). Activated macrophage stage was indicated by white bar (denoted as LPS). Error bars represent technical variation. RQ (relative quantification) = 2^-ddCt^.

Various macrophage populations from different tissues of the body exhibit different morphological and functional phenotypes. Two cytokines, macrophage colony-stimulating factor (M-CSF) and granulocyte-macrophage colony-stimulating factor (GM-CSF), are known to promote differentiation of monocytes into macrophages with distinct phenotypes existing *in vivo*. It is known that M-CSF differentiated macrophages are very similar to peritoneal macrophages [20,21]. As a counterpart of PMA-differentiated U937 cells, M-CSF differentiated macrophages were generated from primary blood monocytes, and further activated with LPS (see materials and methods). Real time PCR shows that both CD14 and CD11b were up regulated with M-CSF differentiated macrophages (Figure 4). Additionally, monocyte-derived macrophages manifest down regulation of TNFalpha in contrast to PMA-differentiated U937 cells where up regulation was shown.

Expression data of 24 top regulated transcriptional regulators from U937 cells including activators, repressors, histone modifying enzymes and histone variants were subjected to cross validation in comparison to primary human monocytes derived macrophages (M-CSF/macrophages). Two distinct groups of genes could be identified (Table 3). Within the first group, 54.2% (n = 13) of the genes (MEF2 family, HLX1, BRI, HDAC5, H2AV, BCL6, EGR1, ID3, IRF9, FOS, Nur77) were differentially expressed during differentiation and/or activation in both U937 cells and primary M-CSF/macrophages. Interestingly, both cell types manifest moderate up regulation (2 ~4 RQ fold) of H2AV. The expression consistency of BCL6, EGR1 and FOS depended upon the stage of cells (either differentiated or activated). Despite the generally known concept that Nur77 is induced only after LPS stimulation, one individual donor manifest up regulation already upon macrophage differentiation. Nevertheless, these factors are most likely to be true regulators in the differentiation and function of monocytic cells. A second group of genes (TCF7L2, HDAC7, EGR2, KLF2, KLF4, FOXA2, OCT2, HES1, PCAF, IRF7, MEF2B) showed a very opposite or no differential gene expression (45.8%; n = 11). Among these factors, TCF7L2 is noteworthy. It is a ubiquitous transcription factor in Wnt signaling pathway, but has not been connected to any immune cell gene transcription up to date.

**Table 3:** Cross validation of 24 transcriptional regulators by real-time PCR

		Ct	ddCt		Ct	ddCt			Ct	ddCt	
		
Ref.Seq	gene	Cont	PMA24	LPS	Mono*	Macro*	LPS*		Mono*	Macro*	LPS*
NM_005587	MEF2A	19.1	-3.2	-2.1	25.2	-2.4	-3	≥	21.2	-1	-1.2
NM_002397	MEF2C	18.9	-3	-2.1	25.5	-2.8	-3	≥	19.8	-1.8	-1.8
NM_005920	MEF2D	25.5	-2.4	-1.6	26.4	-0.7	-1	≈	27.7	-0.8	-1.5
NM_021958	HLX1	22.6	-3.4	-1.8	24.7	-2.1	-2	≈	24.7	-2.2	-2.6
NM_021999	BRI	22.2	-3.1	-1.9	26	-2.9	-3	≈	18.9	-2	-2.8
NM_005474	HDAC5	23.7	-2.3	-2.2	26.8	-2	-2	≈	25.5	-1.8	-2
NM_012412	H2AV	18.6	-1.4	0.1	25.7	-1.1	-2	≈	19	-0.9	-1.3
NM_173158	Nur77	35	-0.2	-1.2	33.4	0	-2	≈	33.5	-1.7	-1.2
NM_001706	BCL6	24.6	-6.3	-5.1	27.8	0.3	-1	≈	20	-0.3	-1.3
NM_001964	EGR1	26.8	-3.9	-0.3	30.5	0.4	-5	≥	22.7	8.1	-2.7
NM_005252	FOS	25.5	-5	-1.4	23.7	2.8	-1	≈	21.6	2.7	-2
NM_002167	ID3	29.6	-3.7	-4.2	31	1.2	1.1	≠	23.5	-2.8	-2.3
NM_006084	IRF9	25.1	-4.5	-2.4	23.9	-1.6	-1	≠	21.9	0.3	-0.1
NM_000399	EGR2	28.2	-2.4	-2.1	27.7	2.6	0	≤	23.8	4.4	-0.7
NM_016270	KLF2	26.4	-5	-6.6	21.7	3	2.4	≈	16.7	5.1	3
NM_021784	FOXA2	29.5	-6.2	-3.7	30.2	3.3	2.2	≈	32.3	3	3
NM_005524	HES1	31.5	-4.8	-6	28	6.9	2.7	≈	21.5	11	9
NM_004235	KLF4	26.6	-3.9	-1.6	22	6.5	3.9	≈	20.3	4.9	4
NM_002698	OCT2	25.5	-3.9	-2.8	22.5	-0.5	-1	≠	19.4	0.8	1
NM_030756	TCF7L2	20.7	-3.1	-2.4	25	-0.4	0	≠	20.7	1.8	1
NM_004030	IRF7	26.4	-3.7	-2.2	25.3	0.1	-1	≠	23.9	1.6	1
NM_014707	HDAC7	22.2	-4.9	-4.2	27.8	1.4	1.1	≠	22.1	-0.3	-0.7
NM_003884	PCAF	25.1	-1.9	1.5	28.1	0.7	1.2	≠	28.4	-0.6	-0.9
NM_005919	MEF2B	27.3	-2.7	1.4	31.9	-0.3	0	≠	27.6	-0.5	-1.3
					Primary cells						
					
		U937 cells			donor 1				donor 2		

* Primary monocytes were isolated from the blood of two healthy individuals designated as donor 1 and donor 2, differentiated by M-CSF into macrophages and stimulated by 10 μg/ml LPS. Degree of individual heterogeneity between two donors was expressed as nearly equal (≈), equal with different propensity (≥ or ≤), and opposite (≠).

### Cellular localization of transcription factors

**Figure 5:**
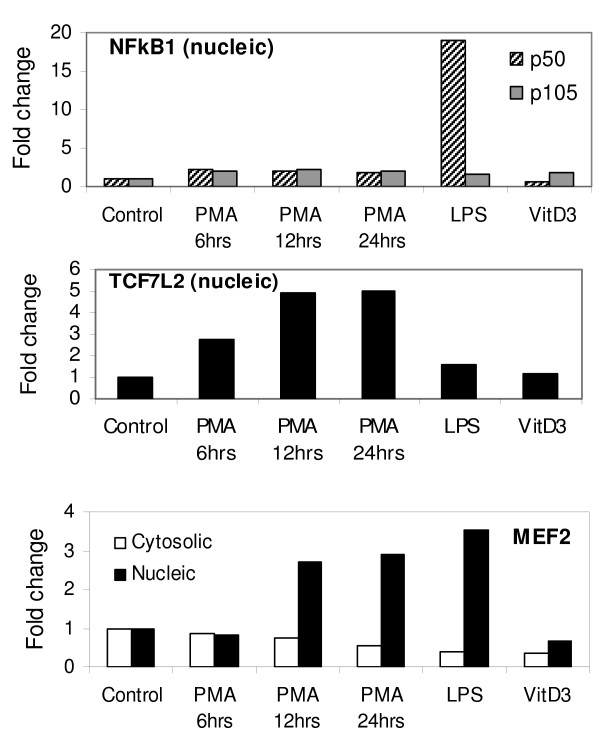
**Protein quantification**. Protein expression of NFkB1/p50, MEF2 and TCF7L2 is induced upon macrophage differentiation and/or LPS stimulation, corresponding to mRNA expression. Prominent induction observed with p50 subunit upon LPS stimulation is in line with known classical role of NFkB1during macrophage activation. TCF7L2 protein was detected only in nucleic fraction. With MEF2 family of proteins, decreasing expression of ca 55 kDa protein was observed (white bar) while nucleic fraction shows increasing expression of ca 60 kDa protein (black bar). The data are normalized to betaActin. Shown is the representative of two independent experiments.

As a step toward verifying actual involvement of selected transcription factors in activating gene program, protein localization was traced and quantified in cytosolic and nucleic fraction by western blot. In order to probe 15 proteins among top regulated transcription factors, twenty different antibodies were tested by ELISA and western blot. Western Blot could detect proteins in cytosolic and nuclear fraction with a clear tendency toward nucleic fraction. Three antibodies were determined to meet the criteria (showing one specific band) that allow their use for protein quantification. Figure 5 shows the results of western blot with these three antibodies. MEF2 antibody recognizes three members (MEF2A, 2C and 2D) simultaneously. One specific band of about 55 kDa in size (white bar in Figure 5, corresponding to the size of MEF2A) was detected in cytosolic fraction while two bands of similar size and intensity (~50 kDa and ~60 kDa, corresponding to MEF2C and 2D) were detected in nucleic fraction (black bar in Fig. 5 represent upper size of ~60 kDa). While expression of cytosolic proteins appears to be decreased, nucleic proteins show 3 ~4 fold induction during macrophage differentiation and subsequent activation by LPS. Up regulation of proteins were comparable to mRNA induction of 3 ~9 RQ fold (ddCt value -1.6 ~-3.2, Table 2). Both NFkB1/p50 and TCF7L2 antibodies produced specific bands with nucleic fractions only. Corresponding to mRNA expression of 5.3 RQ fold (ddCt value -2.4 for LPS sample) and 8.6 RQ fold (ddCt value -3.1 for PMA sample), protein expression of TCF7L2 was clearly induced (up to 5 fold for PMA sample) while LPS sample shows moderate induction of less than 2 fold. Prominent induction of NFkB1/p50 protein after LPS stimulation is also in line with its classical role during inflammatory responses. With all antibodies tested by ELISA, fluorescence detection of antibody binding to protein substrate was observable only with nucleic fraction (data not shown).

## Discussion

### U937 cells as valid model system

The relatively small number of genes up and down regulated after VitD3 differentiation is a strong indication that U937 cells used in this study are nearly proximal to functional monocytes (Figure 2). Despite both CD14 and CD11b are induced in monocyte-derived macrophages, U937 cells-derived macrophages manifest up regulation of CD11b alone (Table 2 and Figure 4). However, lack of CD14 induction should not impair the essential function representing macrophages. CD14 binds LPS to facilitate recognition by TLR4, but does not participate directly in signaling. TLR4 alone can function as a singular signaling receptor for LPS. Moreover, LPS signaling in U937 cells can bypass the necessity of CD14 component of receptor complex due to high concentration of LPS (2 μg/ml or 10 μg/ml) used in this study. The abundance of TNFalpha mRNA prior to differentiation was higher with primary monocytes (data not shown) when compared to U937 cells as manifested by initial Ct value. Taken together, the lack of CD14 expression and the induction of TNFalpha in PMA-differentiated U937 cells can be attributed to the effect of the artificial differentiation system. The MyD88-independent pathway is responsible for sustenance of the proinflammatory program with delayed kinetics of NFkB activation, in the meantime the MyD88-dependent pathway triggers response to initial challenge [22,23]. It is likely that the biphasic behavior of TNFalpha production (up regulation at early and late time points) observed with macrophage-like cells (Figure 1) reflects cross talks and temporal segregation between these two pathways. Up regulation of RB together with p21 upon PMA differentiation is in line with proliferation arrest prior to differentiation process of cells. Both JAK3 and STAT3 (Additional file 3) are known to play a vital role in monocytic differentiation [24,25]. JAK3 (tyrosine kinase) mediates STAT signaling through direct tyrosine phosphorylation of STAT proteins followed by STAT dimerization. Down regulation of MPO, an effector gene of neutrophil function is certainly indicative of macrophage specific gene repression. The presence of Nur77 in the category of LPS inducible genes (Figure 3) is directly corresponding to previous reports in which the role of NR4A orphan receptors (Nur77, Nurr1, Nor1) was described as key regulators of chronic inflammatory diseases thus in the innate immune response [26,27]. Taken together, U937 cells serve a good model system with propensity of differentiation and activation pathways intrinsic to primary monocytes and macrophages.

### Transcriptional regulators involved in macrophages differentiation and activation

Gene expression analysis of transcriptional regulators in both U937 cells and primary macrophages revealed several potentially novel factors. MEF2 family comprises 4 members (MEF2A, B, C, D) that are encoded by different genes. They are described as key regulators in muscle cells and related cell types as in the case of cardiac development [28]. Beside from T cell selection and function [29-31], they are fairly unknown in the context of the immune system. Previously, MEF2C was proved to be activated by p38- mediated phosphorylation to induce transcription of JUN which in turn regulates cytokine gene expression during inflammatory response [32]. More recently, it has been suggested that the MEF2 family is a versatile regulator in diverse cellular systems [33-35]. However, no significant enrichment of MEF2 binding site was seen by promoter analysis of differentially expressed genes (data not shown). Possibly, not all MEF2 binding sites are yet known to be available for complete analysis, thus prevent the final conclusion. On the other hand, MEF2 might act also through differential protein modification and indirect binding to modulate gene expression. Specificity of MEF2 and co-regulator complexes is likely to confer differentiation and immune regulatory function in macrophages. Interestingly, HDAC5 and HDAC9, histone deacetylases, known to form repression complexes with MEF2 family members in many cellular systems are concomitantly up regulated. Both histone deacetylases belong to class II histone deacetylase known to be involved in phosphorylation dependent nucleo – cytoplasmic trafficking in response to activation signals and the formation of repression complexes [36-39]. During muscle development, expression of MEF2 and HDAC9 was shown to be increased during initial phase but diminished expression of HDAC9 at late stage [40]. A likewise operation during macrophage activation mechanism by which MEF2 up regulates its own repressor HDAC9 can be suggested. Among other factors up regulated in both U937 cells and primary macrophages are HLX1 and BRI. HLX1 was described as a marker of immature hematopoietic cells and also known to be involved in the activation of T lymphocyte or NK cells [41,42]. BRI was identified as a LPS-inducible gene in murine macrophages in the presence of CSF-1 [43]. However, there has been no report correlating expression and function of HLX1 and BRI in macrophages. Microarray demonstrated their constant up regulation across different time points of PMA differentiation (Table 2). Additionally, RT-PCR result shows constant up regulation of BRI across LPS stimulation period in U937 cells (data not shown). Taken together, it can be strongly suggested they play a role as effecter molecules of transcriptional reprogramming in macrophages.

### Inconsistent expression pattern and heterogeneity of transcription factors

There are several factors manifesting inconsistent expression patterns between U937 and primary cell systems. TCF7L2 is one of them. On the contrary to PMA/U937 cells with which TCF7L2 induction of both mRNA and protein were verified (Figure 5), M-CSF/macrophages show no induction of TCF7L2 mRNA. Keeping in mind that it is known as the hottest susceptibility gene for type 2 diabetes while being involved in glucose homeostasis, individual heterogeneity of TCF7L2 expression observed with M-CSF/macrophages in this study might be informative. During the past decade, many evidences pointed out type 2 diabetes as a disease of innate immune system, particularly of macrophages, thus closely link the metabolic syndrome to inflammatory pathway [44-46]. Such an inter-relationship promises high probability of TCF7L2 acting in macrophage biology. Another particularly interesting example of inconsistency is up regulation of KLF2 observed with PMA/U937 cells on the contrary to M-CSF/macrophages that shows down regulation. KLF2 was previously reported to be down regulated upon monocyte differentiation or activation in THP-1 cells, and has been proved to be a negative regulator of proinflammatory activation of monocytes [17]. The basis for this difference between U937 and THP-1 cells may be due to the different origin and maturation stage of cells. U937 cells are of tissue origin (histocytic lymphoma) thus at more mature stage. THP1 cells are of blood leukemic origin at less mature stage. Importantly, up regulation rather than down regulation of KLF2 did not produce the consequences of abrogating inflammatory gene activation in U937 cells. Based on the observation of Kumar and colleagues in which p65 and HDACs repress MEF2 transcriptional activation of KLF2 promoter in endothelial cells, it might be reasonable to assume that altered activity balance of antagonistic factors contribute to different cellular activation [47]. The roles of KLF4 and IRF7 in the differentiation or function of macrophages have been also described previously [18,48,49], thus down regulation rather than up regulation with primary macrophages doesn't seem to be immediately accountable. One possible explanation is that an initial amount of protein available in different cell type determine up or down regulation to adjust correct cellular dose. Another explanation is that up regulation can be the effect of U937 cells that are not equal to primary monocytes in the first place. Taken together, the result emphasizes a necessary caution to be exercised when interpreting data obtained from a transformed cell line, alluding differential transcriptional regulatory and signaling pathways between two systems. Nevertheless, the heterogeneity of human individuals observed with several factors (33%; n = 8) including ID3, IRF9, IRF7, TCF7L2, HDAC7/9, OCT2, PCAF and MEF2B raises a possibility that functional capacities of individual macrophages might determine differential susceptibility to and severity of various immune diseases. This enforce the strong argument that investigations using primary cells from different blood donors can greatly impede reliable conclusion of result, thus further emphasize the necessity of a unified model system.

### Potential role of chromatin architecture in macrophage activation

Being surmised that increased expression of genes upon differentiation may be already indicative of their serving important roles in macrophage function afterward, prompt induction of additional 278 genes within 2 hrs of LPS stimulation is intriguing (Figure 2). It might have an implication that promoters of these genes acquired poised status ready to be quickly remodeled upon LPS signal. Accordingly, increased expression of variant histone H2AV, which is also known as H2AZ-2, suggests an exciting possibility. As a dynamic regulator relying on its deposit and loss, incorporation of H2AV in exchange with H2A (canonical histone) could serve as a stable epigenetic mark to keep quiescent promoters in a repressed state while creating more permissive chromatin architecture until appropriate activation signal is received [50-53]. Both forms of protein, H2AZ-1 (previously H2AZ) and H2AZ-2 (previously H2AV), may play new or complementary functions [54]. There are probably several types of chromatin remodeling in general. There may be a large scale remodeling early in development and then more regional remodeling as sections of chromatin become active or are shut down. A more localized type of remodeling takes previously prepared regions and opens them up completely upon receipt of more immediate signals for active transcription. Each type may shares some protein factors and also has unique factor involvement. It is tempting to speculate that identified transcription factors are operating to repress and induce target genes by directing decondensed chromatin structure (H2AV incorporation) during macrophage development which proceed rapid nucleosome remodeling (H2AV eviction) upon LPS stimulation [55]. One possible speculation is whether it functions to activate or silence depend upon the nature of the factors recruited to H2AZ containing nucleosomes while turning off a set of genes and turning on a new subset during the differentiation and inflammatory process.

## Conclusion

### Identification of novel transcriptional regulators

The focus of this study was the identification of transcriptional regulators during macrophage differentiation and activation using U937 cells as a model system. Several novel transcription factors (i.e. MEF2 family, BRI, HLX1, TCF7L2, ID3, NFIL3) were found to be up regulated in addition to those already known (i.e. MAFB, EGR, BCL6, IRF7, NFkB, JUN, FOS, STAT, Nur77) to be involved during macrophage differentiation and/or inflammatory response. We report for the first time both mRNA and protein induction of MEF2 proteins, a MADS-box transcription factor family, during macrophage differentiation and activation in U937 model system. In both U937 cells and primary macrophage cells, three different isoforms of MEF2 (MEF2A, 2C, 2D) appear to be concomitantly induced, thus suggesting functional redundancy of different members. Additionally, MEF2 is directly linking gene activation with epigenetic gene regulation such as histone acetylation and chromatin remodeling [3]. This hypothesis is supported by the fact that interaction partners known to modulate histone modification (HDAC 5 and 9) and chromatin structure (H2AV) are concomitantly expressed.

### Validity of U937 cells as a model system

U937 cells used in this study are proved to be very close to functional monocytes as indicated by the low number of differently expressed genes upon VitD3 differentiation, thus can be an ideal model system to investigate gene regulatory mechanisms in differentiating macrophages. Array analysis of PMA differentiated and LPS activated cells revealed a variety of genes intrinsic to macrophages, inflammation and innate immunity. Nevertheless, how faithfully do these cells resemble primary macrophages is a question to be challenged with carefulness because differentiation pathways in U937 cells-derived macrophages and primary macrophages may diverges to some degree considering the malignant origin of U937 cells. This was asserted by cross validation of 24 transcriptional regulators, which proved a 54.2% (n = 13) of tested factors with consistent expression pattern between PMA differentiated U937 cells and M-CSF differentiated monocytes. The observed differences are based on different macrophage maturation thus highlight the necessity of cross validation. Individual heterogeneity of blood donors further emphasizes the advantage of using a unified model system. Taken together, target genes under investigation should be selected carefully keeping in mind that cells are characterized by how they respond to various stimuli as defined by gene expression. This allows PMA differentiated U937 cells to act as representative of macrophages.

### Future perspectives

As a next step, the role of new identified genes should be verified through functional studies such as RNAi knock down. A further task would be to elucidate how newly identified transcription regulators communicate with factors that modify and remodel chromatin structure of target gene promoters.

## Methods

### U937 cell culture, differentiation, stimulation

U937 cells were maintained in RPMI 1640 medium (Gibco BRL) supplemented with 10% FBS (Biochrome) under humidified air with 5% CO_2 _at 37°C. Cell viability was estimated regularly by trypan blue dye exclusion. To induce differentiation into monocytes or adherent macrophages, the cells were seeded at an initial density of 2 × 10^5 ^cells/ml of VLE-RPMI (Biochrome) and cultured in the presence of 100 nM VitD3 (Calbiochem) or 10 nM PMA (Sigma) for up to 48 hrs at 37°C. Differentiation was monitored by FACS analysis for positive staining of cell surface markers, CD14 and CD11b. Briefly, control and differentiated cells were collected, washed twice in PBS at 4°C, and incubated with 1 μg/ml anti-human FITC-conjugated anti-CD11b and PE-conjugated anti-CD14 (Miltenyi Biotech. Inc.) for 10 min at 4°C and washed once before being resuspended in FACS buffer. For stimulation, differentiated cells were washed, resuspended in VLE-RPMI and stimulated by adding 2 or 10 μg/ml of LPS (Sigma).

### Generation of macrophages from primary blood monocytes

Human white blood cells were separated from blood buffy coat of healthy donors by Ficoll-Paque density centrifugation. To isolate CD14+ monocytes, selection was performed using immunomagnetic particles on AutoMACS (Miltenyi Biotech, Germany) according to manufacturer's instruction. Purity of monocyte suspension was confirmed by cell counting (Sysmex-F 820, Germany), and only preparation proven to have a level of contaminating platelets below the detection limit < 1 × 10^3^/μl was selected. To generate monocyte derived macrophages, 1 × 10^5 ^CD14+ cells were expanded in 96-well flat bottom plates containing 0.2 ml of RPMI 1640 medium with L-glutamine, 10% FCS (PAA laboratories, Austria) and 0.5% penicillin/streptomycin (10000 U/ml, Bayer, Germany). As a differentiation inducer, 50 ng/ml macrophage-colony stimulating factor (M-CSF, Promokine, Germany) was added and cells were cultured for 4 days at 37°C, 5% CO2 atmosphere. LPS stimulation was done at the final concentration of 10 μg/ml.

### RNA isolation, RT-PCR and real-time PCR

Total RNA was extracted from frozen cell pellet by using RNeasy Plus Mini kit (Qiagen) according to the manufacturer's protocol. RNA concentration and purity was measured using NanoDrop 1000A Spectrophotometer. 1 μg of total RNA from each sample was reverse-transcribed to produce cDNA using ImProm-II RT-system (Promega). SyBr-Green (ABgene) real-time PCR was performed with ABI PRISM 7700 to generate 100 ~200 bps of product. All samples were run in duplicate or triplicate and dissociation curve was generated after each run to control formation of primer dimers. GAPDH or betaActin was used as RNA loading control. Normalization of each data point was carried out, in which dCt = Ct_test gene _- Ct_GAPDH_; ddCt = dCt_(test sample) _- dCt_(U937 control)_. Primer sequences are listed in Additional file 4.

### cRNA Hybridization Array

To ensure quantitative accuracy and reproducibility of expression analysis, two independent rounds of differentiation and stimulation experiment were performed. For each experiment, two biological replicates derived from separate culture plates were run in parallel, and represented onto two separate BeadChip slides. Upon RNA isolation and synthesis of labeled cRNA, an additional sample was represented onto a separate slide thus satisfying a technical replicate. Prior to cRNA hybridization, RNA integrity was evaluated by microfluidics analysis using the Agilent 2100 bioanalyzer. cRNA synthesis and labeling was performed using Illumina TotalPrep RNA Amplification Kit (Ambion) then subsequently hybridized, according to the instruction of manufacturer, onto Illumina Sentrix BeadChip Array, HumanRef-8 containing 24,000 features of oligonucleotide probe representing majority of known human genes. Arrays were scanned using Illumina BeadArray reader (V1.7.0.44). Our experimental design adhered to MIAME (Minimum Information About Microarray Experiments) guidelines.

### Differential gene expression analysis

Raw data was acquired as signal intensity of each spot on the array, and normalization was done by variance stabilization method [57]. Signal values of technical or biological replicates were averaged beforehand. Differentially expressed genes were determined based on a comparison of normalized signal intensity ratio between experimental (differentiated and/or stimulated U937 cells) and control (undifferentiated U937 cells) samples, which was expressed as fold change on a spread sheet tables.

### Protein localization and quantification

Protein lysates were prepared using Cytosolic/Nucleic fraction kit (BioCat), and concentrations were determined by Bradford (BioRad) assay. 5 to 10 μg of proteins were run on denaturing 10% polyacrylamide gel after 5 min heating in Laemmli buffer, transferred onto polyvinylidene fluoride (PDVF) membranes (Millipore). After blocking in TBST with 1% BSA for one hour at RT, membrane was incubated overnight at 4°C with primary antibodies (1: 500 or 1: 1,000 dilution). Incubation with horseradish peroxidase conjugated anti-rabbit secondary AB (1: 2,000) was done for 1 hr (RT). Equal amount of protein loading was confirmed by betaActin. After ECL detection, signal intensity of specific band was analyzed by AIDA image analysis program. The NFkB p50/105 polyclonal (H-119) and MEF2 polyclonal antibodies (C-21) were purchased from Santa Cruz Biotech. Monoclonal antibody to TCF7L2 (C9B9, #2565) was from Cell Signalling.

## Authors' contributions

YB and HS were primarily responsible for the design, coordination, conduct of the study and drafted the manuscript. SH were responsible for array normalization and bioinformatic analysis. HH and GB provided U937 cells and primary macrophages. MHS contributed to experimental design of U937 cell system. SS and HL contributed editorial revisions, provided comments and discussion. All authors read and approved the final manuscript.

## Supplementary Material

Additional file 1 **Correlation coefficients calculated from microarray experiments (Illumina)**. Each of biological replicates (A and B) and technical replicates (A1 and A2) were represented onto separate BeadChip slides (Slide 1, 2, 3) and hybridized with untreated U937 cells, PMA 6 hrs, 12 hrs, 24 hrs, 32 hrs, LPS and VitD3 samples. Click here for file

Additional file 2 **Complete list of gene expression data (containing 20,588 features)**. Array fold change, sorted by LPS sample, reflects logarithmic scale upon normalization by 'stabilization method' (Weber et al. 2002). Click here for file

Additional file 3 **Complete list of 709 transcription factor gene expression**. Array fold change, sorted by LPS sample, reflects logarithmic scale upon normalization by 'stabilization method'. Click here for file

Additional file 4 **List of primers**. Shown are sequences of 61 primer sets used for real time PCR. Click here for file
